# Psychological Challenges Among Older Adults with Early Stage Dementia: A Narrative Literature Review

**DOI:** 10.1093/geroni/igab046.2758

**Published:** 2021-12-17

**Authors:** Mijin Jeong

**Affiliations:** University of Kansas, LAWRENCE, Kansas, United States

## Abstract

Due to rapid expected growth in the population of older adults with dementia, exploring the issues and experiences related to early stage dementia (ESD) is a fundamental step toward helping individuals adjust to their diagnosis and transition into treatment. The purpose of this paper is to review the extant literature regarding how older adults adjust to and cope with the onset of dementia through major situations and difficulties. A narrative approach was applied to review 120 articles focused on ESD that were published in the U.S. and other western countries between 1995 to 2020. There were four apparent themes in the literature, which align with key chronological experiences related to ESD: diagnosis of dementia; stigma related to dementia; the development of identity with ESD; and social and service-related experiences of older adults with ESD. Stigma related to dementia was a powerful risk factor that hindered psychological adjustment to ESD. Varied cultural perspectives on dementia and a lack of knowledge of dementia symptoms among diverse older adults and their families were also major risk factors. In the U.S., there was a lack of literature, especially around the development of identity with dementia and older adults’ perspectives on available services, Also, there were insufficient U.S.-based studies that explored the challenges of psychological adjustment among racial and ethnic minority groups. Future research could benefit from taking a life course perspective to assess ESD within the context of one’s life and examine challenges associated with ESD across all four themes to promote empowerment.

